# Supplementary figures and images for: Geometric morphometrics of endophytic oviposition traces of Odonata (Eocene, Argentina)
Source: R Soc Open Sci. 2020 Dec 16;7(12):201126. doi: 10.1098/rsos.201126 (PMC7813221; doi:10.1098/rsos.201126)

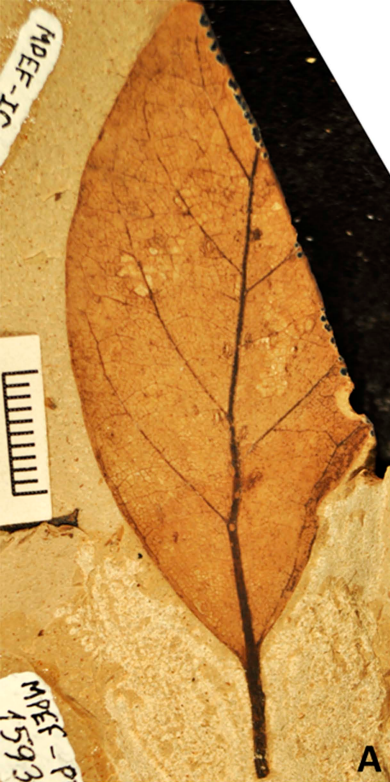

A

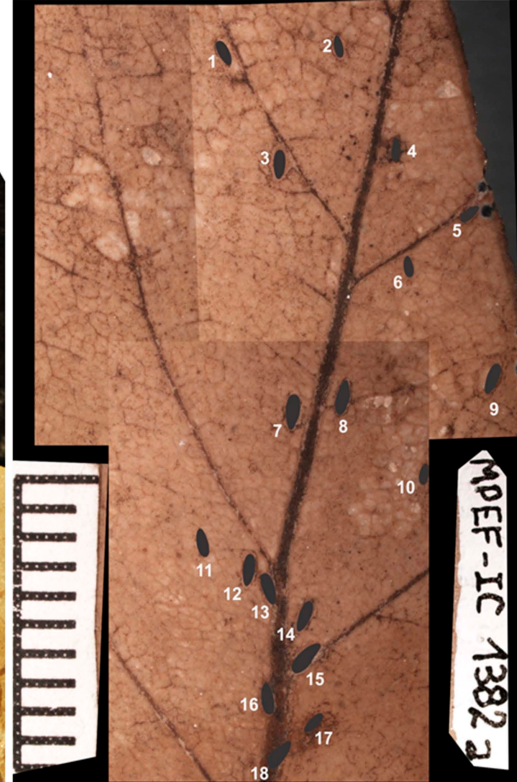

B

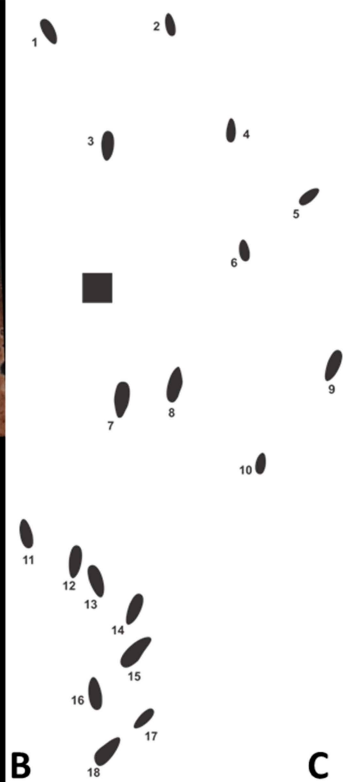

C

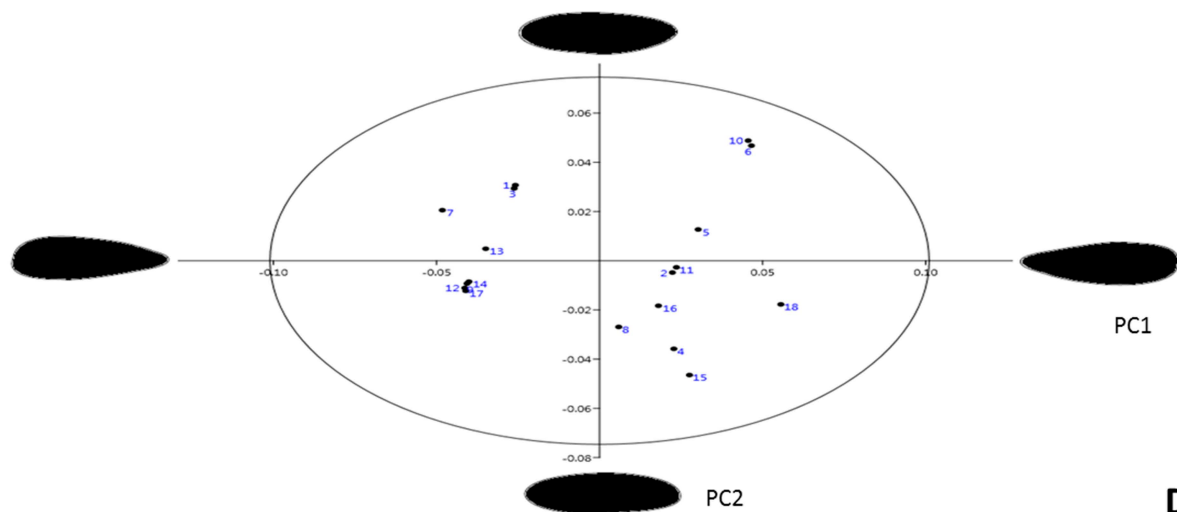

D

Supplement: Material MPEF-IC-1382 [file rsos201126supp1.pdf]

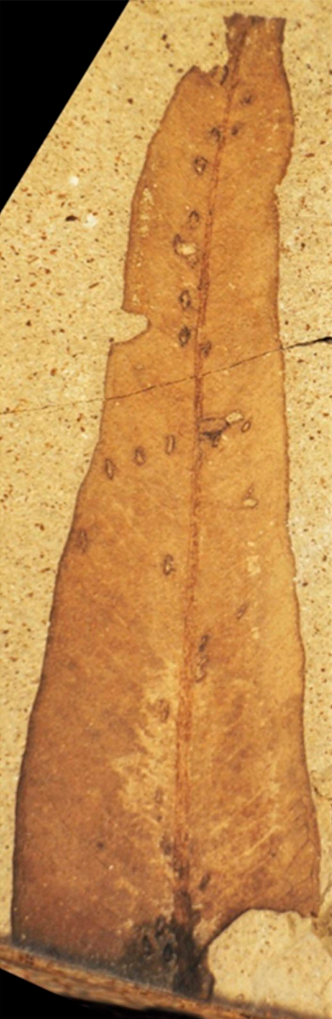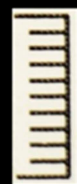

MPEF-TC 1376

**A**

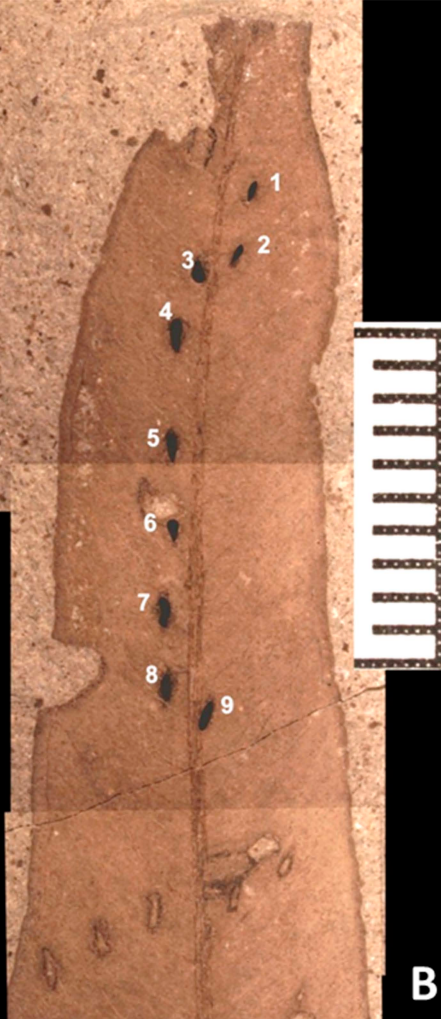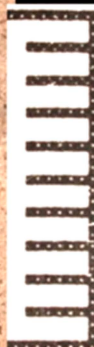

**B**

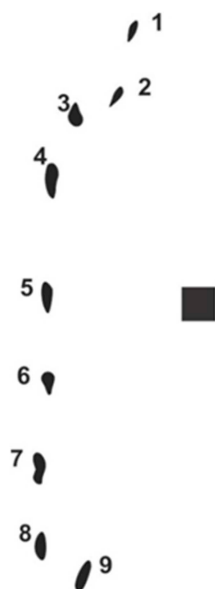

**C**

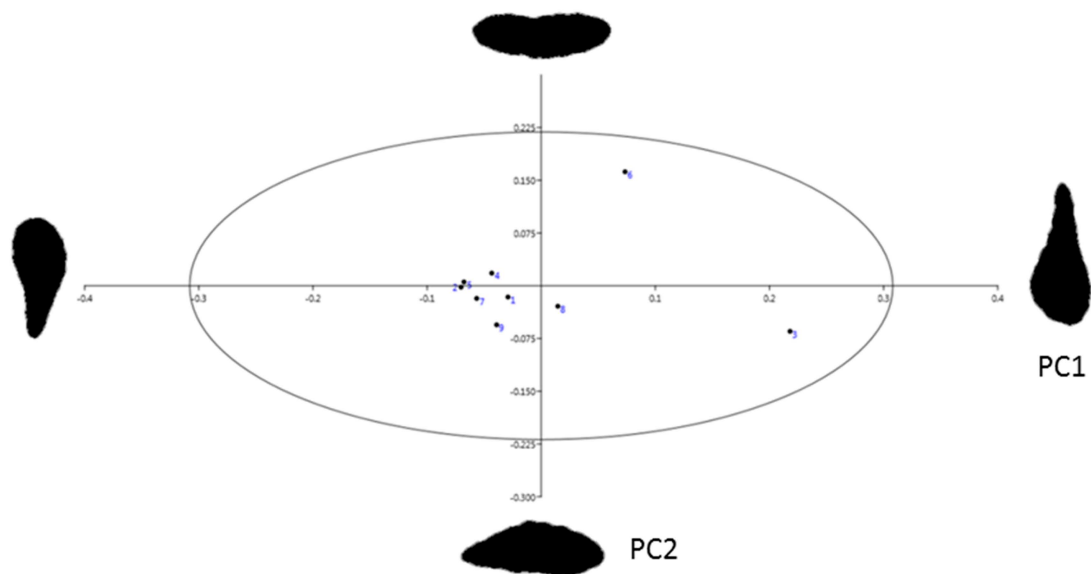

**D**

Supplement: Material MEF-IC-1376 [file rsos201126supp2.pdf]

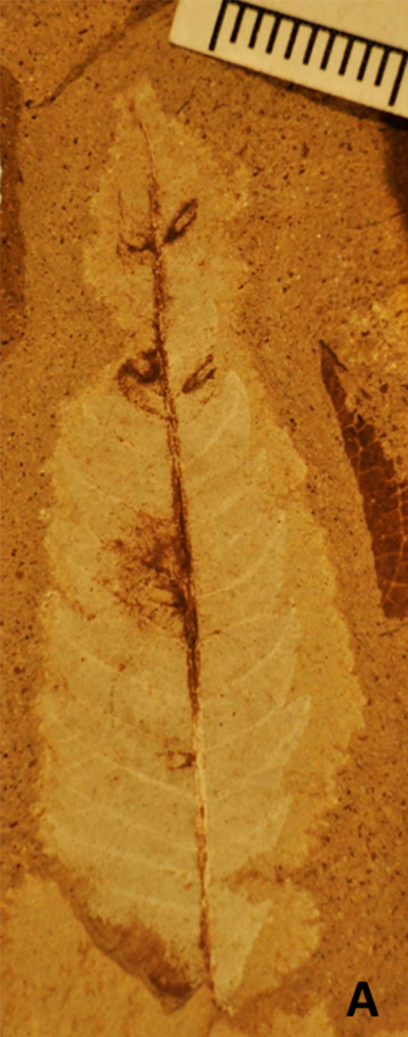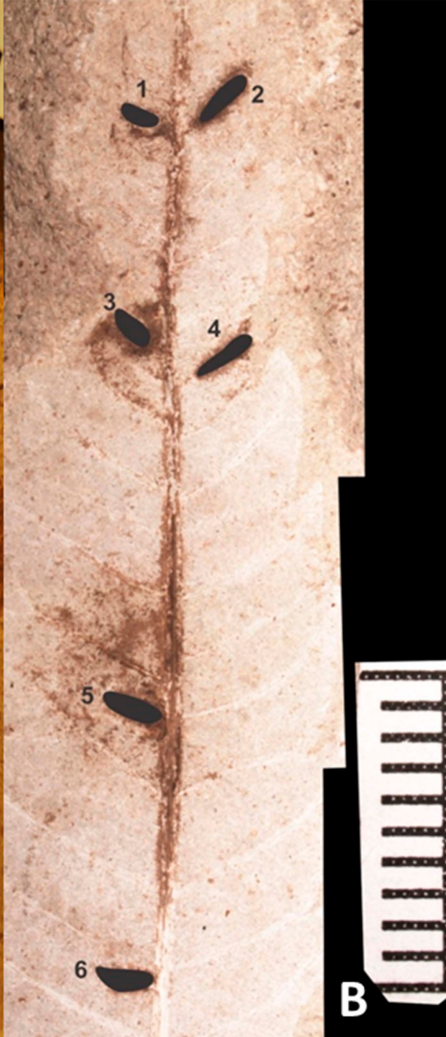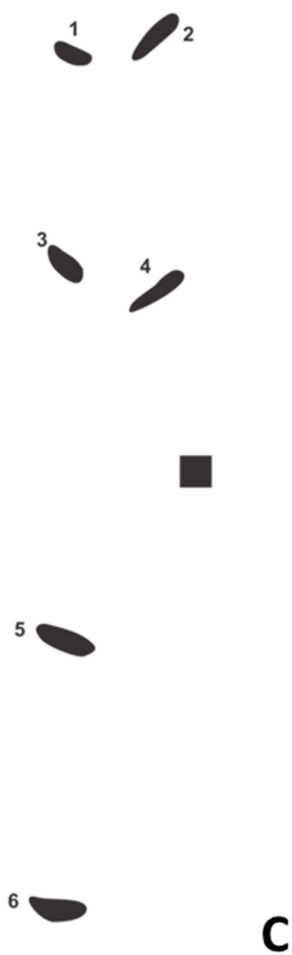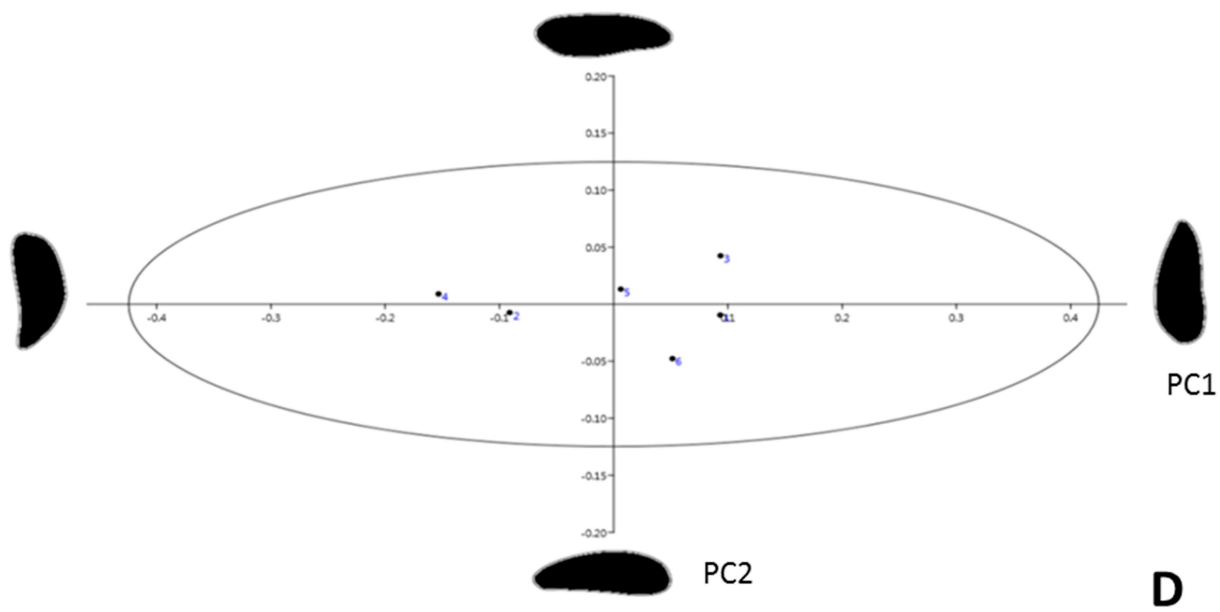

Supplement: Material MEF-IC-1385 [file rsos201126supp3.pdf]

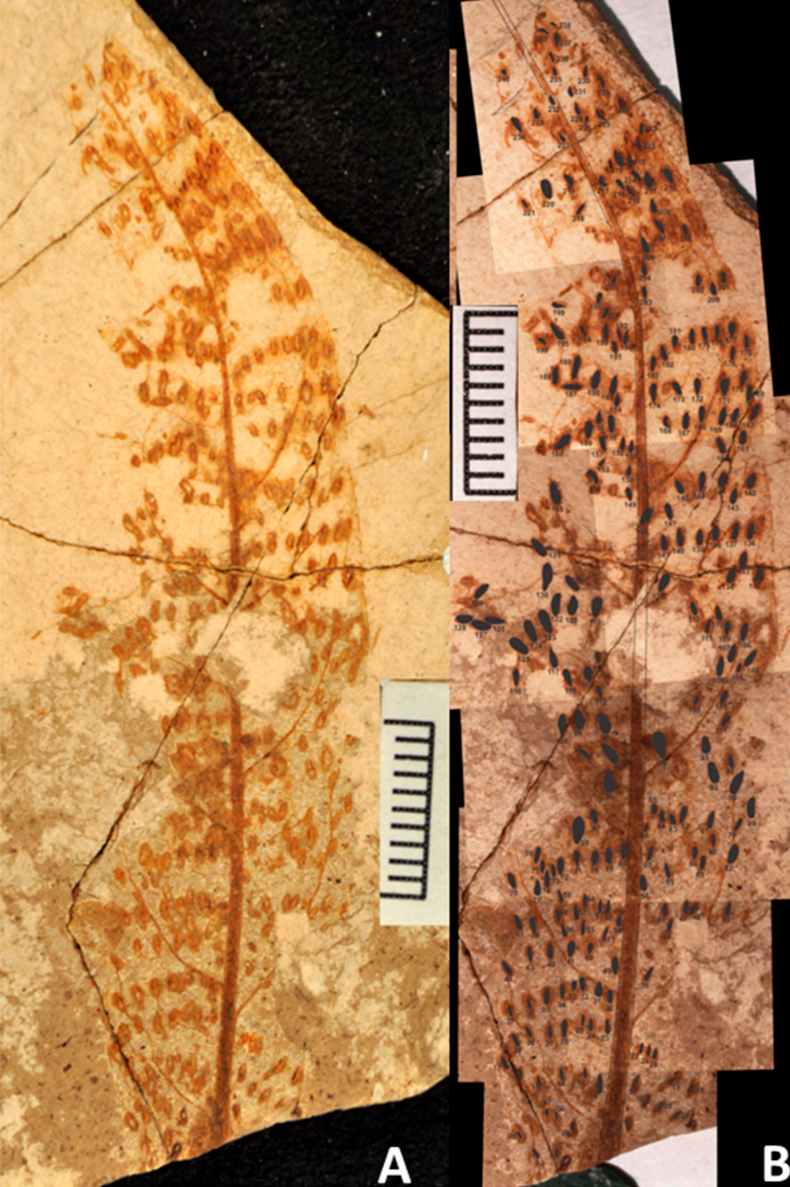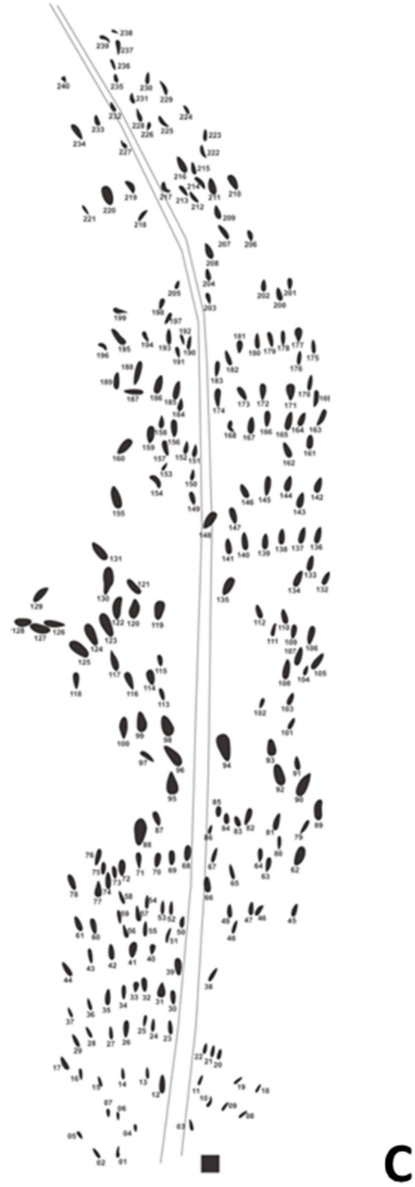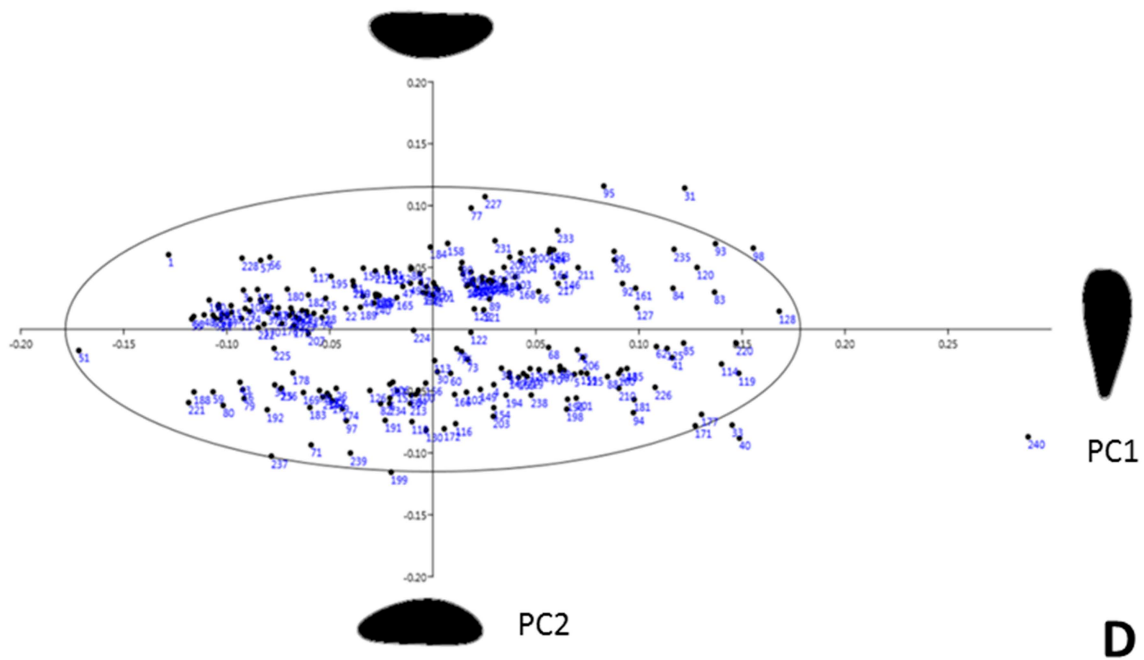

Supplement: Material MEFP-IC-1388 [file rsos201126supp4.pdf]

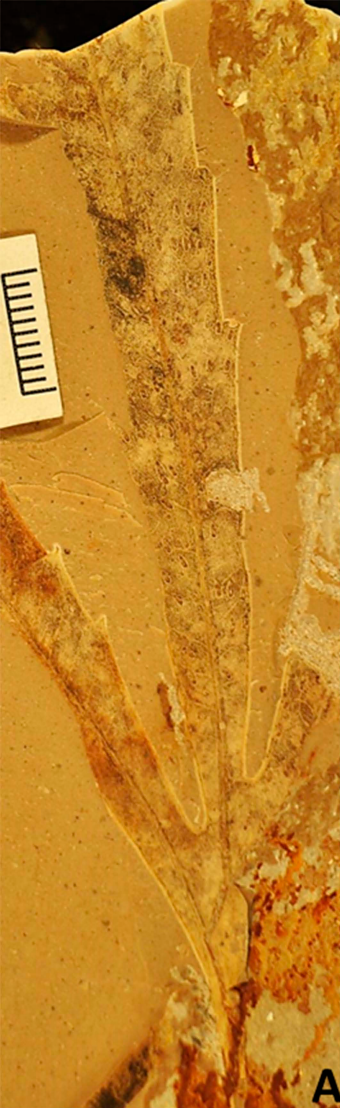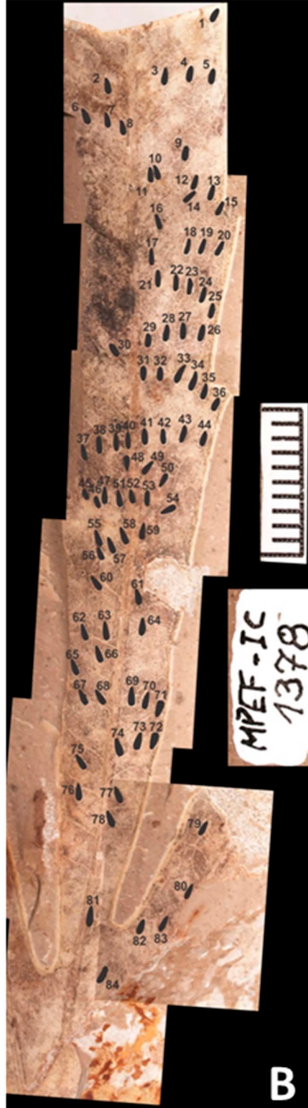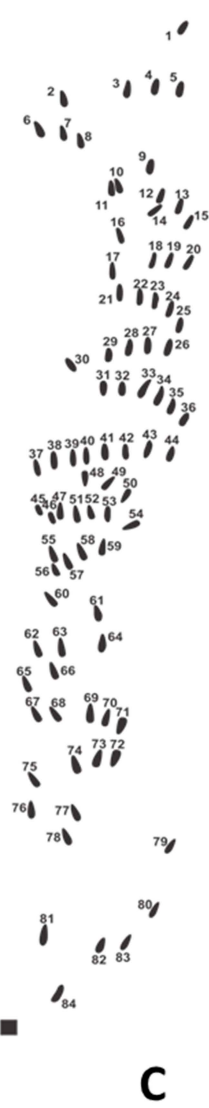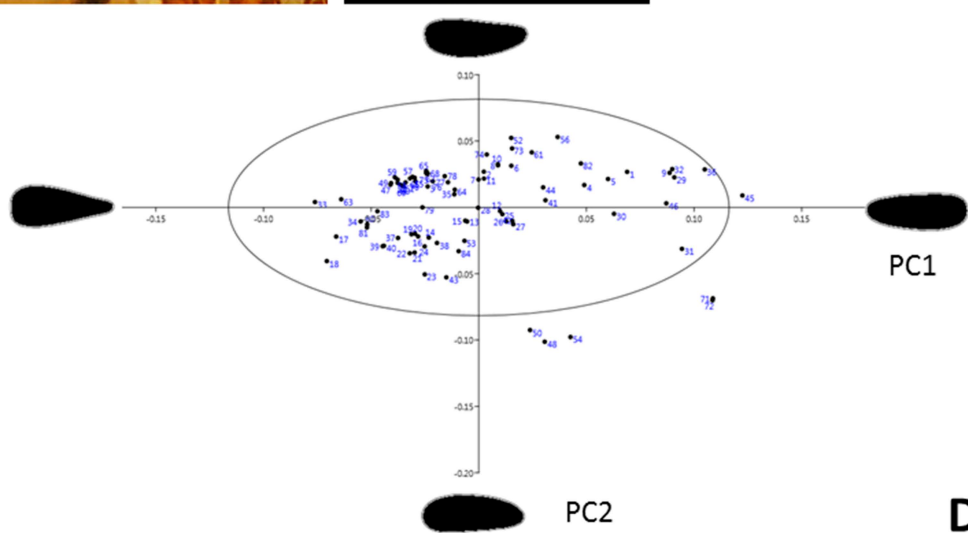

Supplement: Material MEF-IC-1378 [file rsos201126supp5.pdf]
